# Quantitative and Sensitive Detection of *GNAS* Mutations Causing McCune-Albright Syndrome with Next Generation Sequencing

**DOI:** 10.1371/journal.pone.0060525

**Published:** 2013-03-25

**Authors:** Satoshi Narumi, Kumihiro Matsuo, Tomohiro Ishii, Yusuke Tanahashi, Tomonobu Hasegawa

**Affiliations:** 1 Department of Pediatrics, Keio University School of Medicine, Tokyo, Japan; 2 Department of Pediatrics, Asahikawa Medical University, Hokkaido, Japan; Children's National Medical Center, United States of America

## Abstract

Somatic activating *GNAS* mutations cause McCune-Albright syndrome (MAS). Owing to low mutation abundance, mutant-specific enrichment procedures, such as the peptide nucleic acid (PNA) method, are required to detect mutations in peripheral blood. Next generation sequencing (NGS) can analyze millions of PCR amplicons independently, thus it is expected to detect low-abundance *GNAS* mutations quantitatively. In the present study, we aimed to develop an NGS-based method to detect low-abundance somatic *GNAS* mutations. PCR amplicons encompassing exons 8 and 9 of *GNAS*, in which most activating mutations occur, were sequenced on the MiSeq instrument. As expected, our NGS-based method could sequence the *GNAS* locus with very high read depth (approximately 100,000) and low error rate. A serial dilution study with use of cloned mutant and wildtype DNA samples showed a linear correlation between dilution and measured mutation abundance, indicating the reliability of quantification of the mutation. Using the serially diluted samples, the detection limits of three mutation detection methods (the PNA method, NGS, and combinatory use of PNA and NGS [PNA-NGS]) were determined. The lowest detectable mutation abundance was 1% for the PNA method, 0.03% for NGS and 0.01% for PNA-NGS. Finally, we analyzed 16 MAS patient-derived leukocytic DNA samples with the three methods, and compared the mutation detection rate of them. Mutation detection rate of the PNA method, NGS and PNA-NGS in 16 patient-derived peripheral blood samples were 56%, 63% and 75%, respectively. In conclusion, NGS can detect somatic activating *GNAS* mutations quantitatively and sensitively from peripheral blood samples. At present, the PNA-NGS method is likely the most sensitive method to detect low-abundance *GNAS* mutation.

## Introduction

The rapid emergence of next generation sequencing (NGS) is revolutionizing medical sciences. NGS now allows clinical investigators to analyze transcriptome, exome and genome from small amounts of DNA/RNA. NGS is also available for ultra-deep sequencing of PCR amplicons, microRNA and microbiomes. NGS-based approaches have brought remarkable advances in a broad range of medical research areas, such as studies of rare Mendelian disorders [1] and surveillance of infectious disease outbreaks [2]. NGS has also provided a wealth of new information for cancer genomics, owing in part to the ultra-deep amplicon sequencing of cancerous and precancerous cells [3]. Because NGS can analyze millions of DNA fragments simultaneously and independently, low abundance mutations of oncogenes have now become readily detectable. However, in contrast to advances in understanding of somatic mutations associated with cancer, knowledge about somatic mutations causing benign congenital disorders remains very limited.

McCune-Albright syndrome (MAS; OMIM #174800) is a rare congenital disorder hallmarked by osseous fibrous dysplasia, café-au-lait skin pigmentation and various endocrine hyperfunction, *e.g.,* peripheral precocious puberty, Cushing syndrome and functional pituitary adenoma [4], [5]. MAS is caused by activating mutations of *GNAS*, encoding the stimulatory G-protein alpha subunit [6]. Mutations are exclusively present in the somatic mosaic state, probably because the nonmosaic state leads to early embryonic lethality. Clinical manifestations of MAS are highly variable in all three lesions, presumably due to variability of mutation abundance among affected tissues.

In MAS patients, mutation abundance is generally low in unaffected tissues. Thus, mutations in peripheral blood leukocytes (PBL) cannot be detected by standard PCR-based Sanger sequencing, while mutations in affected lesions (*e.g.,* surgical bone specimens) can. Based on the fact that the vast majority of activating *GNAS* mutations occurs in the Arg201 residue, Candeliere *et al.* developed the method for selective enrichment of Arg201 *GNAS* mutations using a series of nested PCR and restriction enzyme digestion [7]. Subsequently, the second enrichment method with use of a peptide nucleic acid (PNA) probe, which forms hybrids with wildtype DNA and inhibits PCR amplification, was developed [8]. Mutation detection rate from PBL samples with these two methods are typically around 50% [9]–[11]. Of interest, mutation detection rate increases up to 90% when DNA sample derived from the affected lesion is available [10]. This implies that diagnostic performance of the two methods is still inadequate.

In the present study, we developed a novel NGS-based method that can detect low-abundance *GNAS* mutations quantitatively and sensitively. We compared diagnostic performance of the NGS-based method with that of the PNA method, by a serial dilution study and a mutation detection study using 16 MAS patient-derived PBL samples.

## Materials and Methods

### PCR with or without the PNA probe

**Figure 1:**
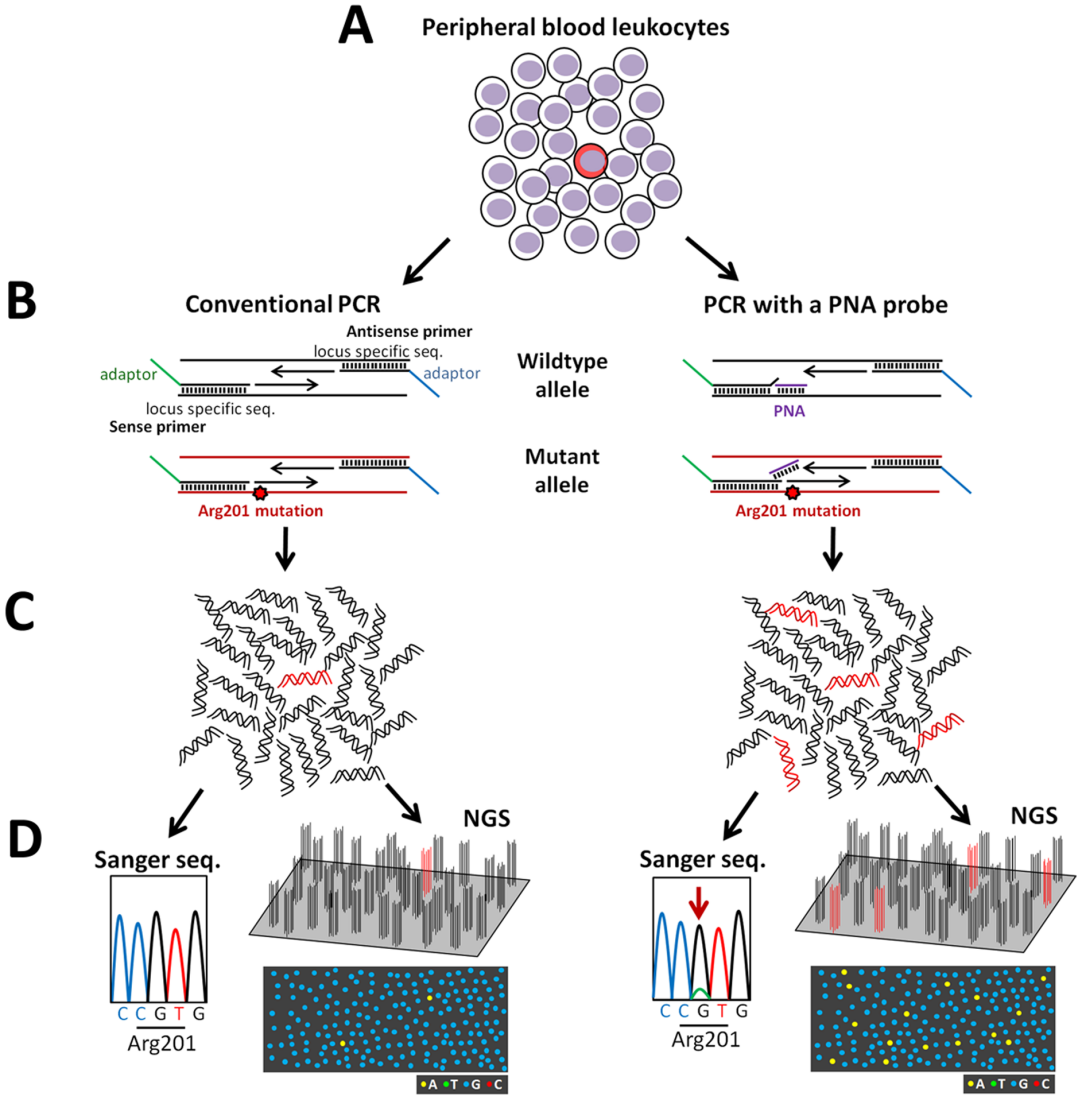
Schematic diagramas showing an overview of mutation detection methods. (**A**) In patients with McCune-Albright syndrome, the proportion of mutation-carrying cells (colored red) is low in peripheral blood leukocytes (PBL). (**B**) In the present study, PCR amplification was conducted in the absence (*left panel*) or presence (*right panel*) of the peptide nucleic acid (PNA) probe. The PNA probe preferentially hybridizes to wildtype PCR fragments (colored black) and inhibits their amplification. This results in enrichment of mutant PCR fragments (colored red). We used chimeric PCR primers, containing both locus-specific and adapter sequences, to generate amplicons that are sequenced on the Illumina platform. (**C**) PCR without the PNA probe produces PCR amplicons, of which relative proportion between wildtype (colored black) and mutant (colored red) is similar to PBL (*left panel*). In contrast, PNA treatment enriches mutant amplicons (*right panel*). (**D**)**,** PCR amplicons were analyzed by both Sanger sequencing and next generation sequencing (‘NGS’). Due to low mutation abundance, mutations cannot be detected in amplicons generated without the PNA probe (*left panel*), while they can be detected in PNA-treated amplicons (*right panel,* an arrow indicates the mutation). In the MiSeq platform, clonal clusters, each derived from a single DNA molecule, are generated on a flow cell, and are sequenced base-by-base simultaneously and independently. The diagrams under the schematic flow cells show imaginative optically scanned data of the cycle corresponding to the mutated nucleotide. In a sample without PNA treatment, the mutant amplicons can be recognized on the flow cell (*left panel*). Mutant-enriched samples are also analyzable by NGS (*right panel*).

The overview of mutation detection methods is shown in **Figure 1**. All DNA samples used in the study was extracted from PBL with the Gentra Puregene Blood Kit (Qiagen, Hilden, Germany). Partial region of the *GNAS* locus (chr20:57484398-57484647; hg19), in which nucleotides 598 to 711 (begins at the first ATG codon) were included, was PCR-amplified with or without the PNA probe. The PCR mixture (final volume 20 µL) contained 100 ng genomic DNA, 0.25 mM dNTPs, primers (0.25 µM each), and 1 U Herculase II Fusion DNA Polymerases in reaction buffer (Agilent Technologies, Santa Clara, CA), with or without 30 µM PNA probe (Panagene Inc., Daejeon, Korea). The PCR conditions were as follows: initial denaturation at 98°C for 30 s; 35 cycles at 98°C for 10 s (denaturation), 68°C for 60 s (hybridization), 55°C for 30 s (annealing) and 72°C for 30 s (extension) with a final extension at 72°C for 5 min. The sequences of the PNA and primers were as follows: PNA Gly-NH_2_-CGC TGC CGT GTC-HAc; sense primer 5′-CTA CAC GAC GCT CTT CCG ATC TGT TTC AGG ACC TGC TTC GC-3′; and antisense primer 5′-GTG ACT GGA GTT CAG ACG TGT GCT CTT CCG ATC TCA CAG CAT CCT ACC GTT GAA-3′ (adaptor sequences used in Illumina platform are underlined). Generated PCR products were purified with the Agencourt AMPure XP Bead system (Beckman Coulter Genomics, Essex, UK), and were subject to both Sanger sequencing and NGS.

### Mutation detection by Sanger sequencing and NGS

For Sanger sequencing, we used the BigDye Dideoxy Sequence Kit (Life Technologies, Carlsbad, CA) and the ABI3130*xl* sequencer (Life Technologies). Presence/absence of mutations was judged based on visual inspection of each sequence chromatogram.

As for NGS, we performed 15 cycles of second PCR using diluted first PCR products to add the attachment sites (P5 and P7) and the index sequence, which are used in Illumina multiplexed sequencing. The PCR mixture (final volume 20 µL) contained 1 µL purified first PCR product (diluted 1∶20 with pure water), 0.25 mM dNTPs, primers (0.25 µM each), and 1 U Herculase II Fusion DNA Polymerases in reaction buffer. The PCR conditions were as follows: initial denaturation at 98°C for 30 s; 15 cycles at 98°C for 10 s, 55°C for 20 s and 72°C for 30 s with a final extension at 72°C for 5 min. The sequences of the primers were as follows: sense primer: 5'- AAT GAT ACG GCG ACC ACC GAG ATC TAC ACT CTT TCC CTA CAC GAC GCT CTT CCG ATC T-3'; and antisense primer: 5'- CAA GCA GAA GAC GGC ATA CGA GAT NNN NNN GTG ACT GGA GTT CAG ACG TGT-3' (P7 and P5 attachment sites are underlined. NNN NNN in the antisense primer denotes index-specific sequence). The second PCR products were purified with the AMPure system, and were quantified with the Qubit dsDNA HS Assay Kit (Life Technologies). In each NGS run, 16 samples were multiplexed per pool. To improve base call accuracy, an equimolar quantity of the PhiX control (Illumina, San Diego, CA) was added to the pool.

Pooled samples were pair-end sequenced on the MiSeq instrument with at least 30 cycles and an index read. Base calling, read filtering and demultiplexing were performed according to the standard Illumina processing pipeline. Sequence reads were mapped to the *GNAS* genomic sequence with Bowtie [12]. We used SAMtools to calculate read depth and nucleotide frequencies for each position of the amplicons [13]. Filtering threshold was set to Q35, which is equivalent to the probability of an incorrect base call 1 in 3160 times. For each experiment, three control PBL DNA samples were analyzed to define the experiment-specific reference upper limit of the variant call (z-score equal or more than 2.5 were defined as positive). All DNA samples were amplified and sequenced twice.

### Serial dilution of cloned mutant DNAs

A first PCR product generated from an R201H mutation carrying patient was cloned into the pCR2.1-TOPO vector (Life Technologies). We prepared wildtype or mutant DNA (each 1 ng/ µL) by diluting sequence-verified plasmids. Then, we diluted cloned mutant DNA into cloned wildtype DNA to 1/10 [relative mutation abundance (RMA), 10%], 1/100 (1%), 1/333 (0.3%), 1/1,000 (0.1%), 1/3,333 (0.03%) and 1/10,000 (0.01%). Serially diluted DNA samples were subject to sequence analyses described above.

### Clinical samples

In a comparative mutation detection study, 16 PBL genome samples derived from MAS patients (6 boys and 10 girls) were used. The 16 patients had classic form of MAS with two or three features of the triad (osseous fibrous dysplasia, café-au-lait skin pigmentation and endocrine hyperfunction) (**Table 1**). Fibrous dysplasia, café-au-lait skin pigmentation and endocrine hyperfunction were observed in 14 (88%), 12 (75%) and 14 (88%), respectively. Eight subjects (50%) had all three features. Observed endocrine dysfunction includes peripheral precocious puberty (N = 9), functional thyroid adenoma (N = 3), functional pituitary adenoma (N = 2) and Cushing syndrome (N = 2) (**Table 1**).

**Table 1:** Characteristics of the study subjects.

ID	Sex	MAS features			Relative mutation abundance (%)	Mutation detection method		
		FD	Skin lesion	Endocrine hyperfunction		PNA	NGS	PNA-NGS
1	F	Present	Present	Peripheral PP	12.4	R201C	R201C	R201C
2	M	Present	Present	Functional pituitary adenoma*, Functional thyroid adenoma	4.2	R201C	R201C	R201C
3	M	Present	Absent	Cushing syndrome	3.4	R201H	R201H	R201H
4	F	Absent	Present	Peripheral PP	2.9	N.D.	R201C	R201C
5	F	Present	Present	Peripheral PP	1.4	R201H	R201H	R201H
6	M	Present	Present	Absent	0.81	R201H	R201H	R201H
7	M	Present	Present	Cushing syndrome	0.67	R201H	R201H	R201H
8	F	Present	Present	Peripheral PP, Functional thyroid adenoma	0.55	R201H	R201H	R201H
9	M	Present	Present	Functional thyroid adenoma	0.28	R201H	R201H	R201H
10	M	Present	Absent	Pituitary adenoma**	0.26	R201C	R201C	R201C
11	F	Present	Present	Peripheral PP	<0.03	N.D.	N.D.	R201C
12	F	Present	Present	Peripheral PP	<0.03	N.D.	N.D.	R201H
13	F	Absent	Present	Peripheral PP, Functional thyroid adenoma	<0.03	N.D.	N.D.	N.D.
14	F	Present	Absent	Peripheral PP	<0.03	N.D.	N.D.	N.D.
15	F	Present	Absent	Peripheral PP	<0.03	N.D.	N.D.	N.D.
16	F	Present	Present	Absent	<0.03	N.D.	N.D.	N.D.

Abbreviations: FD, osseous fibrous dysplasia; MAS, McCune-Albright syndrome; N.D., not detected; NGS, next generation sequencing; PNA, the peptide nucleic acid method; PNA-NGS, combinatory use of PNA and NGS; PP, precocious puberty

* Hyperprolactinemia and GH-producing adenoma

** GH-producing adenoma

### Ethics statement

The study was approved by the Institutional Review Boards of Asahikawa Medical University and Keio University School of Medicine. Written informed consent for molecular studies was obtained from the subjects or his/her parents.

## Results

### GNAS amplicon sequencing by NGS

We designed chimeric primer pairs, containing both locus-specific and adapter sequences, to generate PCR amplicons that are directly sequenced on the Illumina MiSeq platform. The amplicon covers two known sites of activating *GNAS* mutations (*i.e.,* Arg201 and Gln227 [14]), thus would be expected to detect most mutations causing MAS. This experimental design allowed us to generate very high read depth per sample (approximately 100,000) with low error rate (mean±SD, 0.011±0.005%) (data not shown).

### Quantitative detection of a GNAS mutation

**Figure 2:**
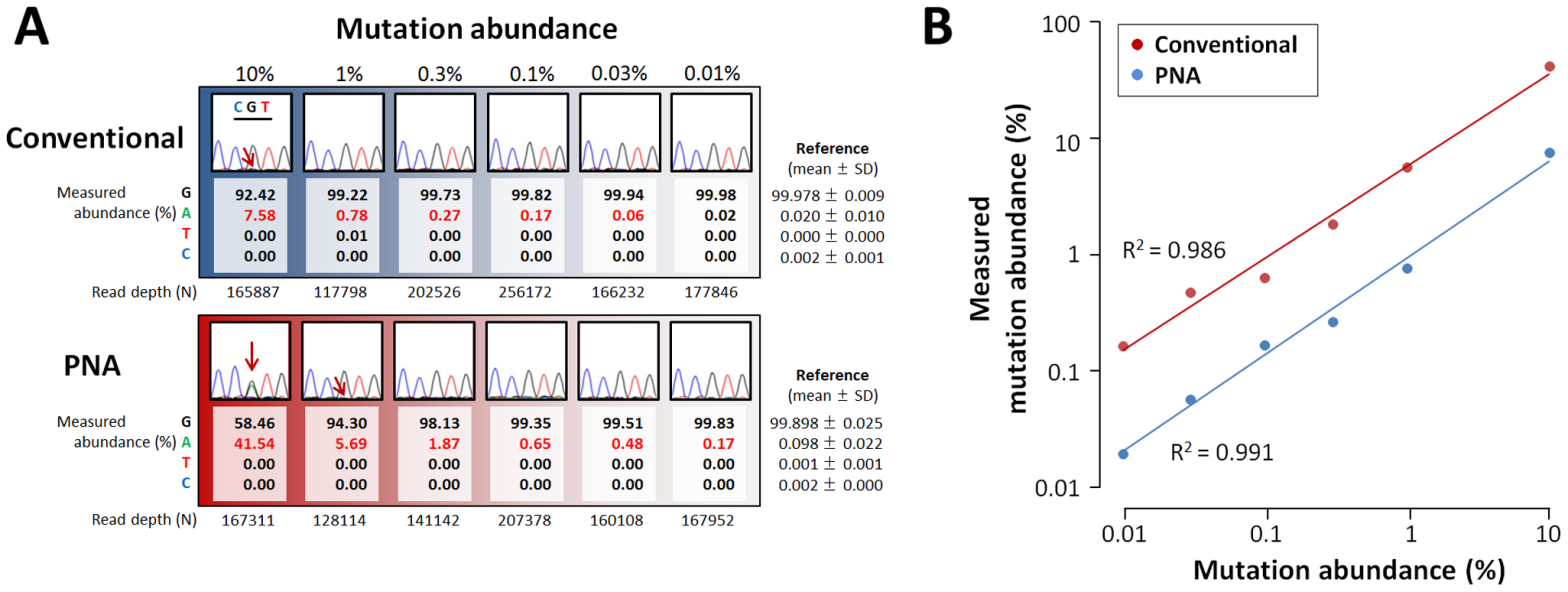
Results of the serial dilution study. (**A**) Cloned mutant DNA (R201H mutation) was diluted into cloned wildtype DNA to 1/10 (10%), 1/100 (1%), 1/333 (0.3%), 1/1,000 (0.1%), 1/3,333 (0.03%) and 1/10,000 (0.01%). Serially diluted DNA samples were PCR-amplified with or without the peptide nucleic acid (PNA) probe (‘PNA’ and ‘Conventional’. Each PCR product was analyzed by Sanger sequencing and next generation sequencing (NGS). Partial chromatograms encompassing the GNAS codon 201 (indicated by CGT) are shown. Relative abundance of the c.602 nucleotide (G, A, T and C) measured by NGS is aligned with each chromatogram. The G allele is wildtype, while the A allele is the R201H mutant. Values with a positive test result (defined by z-score of measured mutant abundance; see Materials and Methods for details) are colored red. Experiment-specific reference ranges are also shown. In the 12 chromatograms, the mutant signal could be detected in two PNA-treated samples and one non-treated sample (indicated by red arrows). Contrastingly, the mutation could be detected down to 0.03% by NGS alone, and down to 0.01% by combinatory use of PNA and NGS. (**B**) A serial dilution plot showing a linear correlation between true mutation abundance and measured mutation abundance. Note that both axes are logarithmic. Comparison of mutation abundance values of PNA-treated and untreated samples revealed that the fold enrichment by PNA is about 7, and it was independent of initial abundance.

To test the ability of the NGS-based mutation detection to provide quantitative data, we conducted a serial dilution study using cloned plasmid DNA samples (wildtype or R201H). We serially diluted mutant DNA into wildtype DNA, and measured mutant abundance with NGS. As we expected, a linear correlation between true mutant abundance and measured relative mutation abundance (NGS-measured RMA; defined as the proportion of sequence reads containing the mutation), was observed down to 0.01% (**Figure 2**), indicating reliable quantification.

### Mutation abundance in PBL of MAS patients

To define the distribution of NGS-measured RMA in PBL among MAS patients, we analyzed 16 patient-derived PBL samples. Ten out of 16 genomes had a *GNAS* mutation (R201H, N = 6; R201C, N = 4) of which NGS-measured RMA was more than 0.03% (**Table 1**). NGS-measured RMA ranged from 0.3% to 12.4% (median, 1.1%), which was consistent with the fact that we could not detect those mutations by conventional Sanger sequencing without the PNA probe (data not shown). It is also consistent with the previous genetic knowledge that conventional Sanger sequencing cannot detect *GNAS* mutations in most MAS patient-derived PBL samples [7]. The distributions of NGS-measured RMA were similar between eight patients having all three MAS features and the remaining eight with two features (P = 0.5, Wilcoxon rank sum test).

### Comparison of mutation detection methods

Finally, we compared the diagnostic performance of three mutation detection methods: the PNA method, NGS, and combinatory use of PNA and NGS (PNA-NGS). We assessed the detection limits using the serially diluted samples, and found that NGS could detect the mutation down to 0.03%, while the PNA method alone could detect down to 1% (**Figure 2**). The PNA-NGS method had the lowest detection limit, which was 0.01% (**Figure 2**). We also performed a comparative mutation detection study using the 16 patient-derived PBL genome samples. The PNA method identified mutations in nine out of 16 patients (**Table 1**). All of these mutations were also detected by NGS and PNA-NGS. Among seven patients with a negative result by the PNA method, NGS detected one mutation carrier, and PNA-NGS revealed further two mutation carriers (**Table 1**). Collectively, mutation detection rate of PNA, NGS and PNA-NGS was 56%, 63% and 75%, respectively.

## Discussion

Detecting low-abundance somatic mutations with next generation amplicon sequencing is becoming a robust analytic tool in cancer genomics. In the present study, we demonstrate that this approach is also effective in diagnosis of a benign disorder due to low-abundance somatic mutations, as shown in megalencephaly syndromes very recently [15].

The quantitative nature of NGS allowed us to investigate the distribution of RMA in patient-derived PBL samples. We showed for the first time that NGS-measured RMA in PBL is strikingly variable among MAS patients. NGS-measured RMA in PBL does not correlate with disease severity, as defined by the number of clinical features, indicating that RMA in PBL and affected lesions are not correlated. Similar results have been observed in syndromes due to activation of AKT signaling (Proteus syndrome [16], and megalencephaly syndromes [15]), thus would be a universal feature of congenital syndromes due to somatic activating mutations.

We verified the diagnostic performance of three mutation detection methods (PNA, NGS and PNA-NGS) using a serial dilution study and a comparative mutation detection study. In both studies, the PNA-NGS method was seemed to be most sensitive. Combination of NGS and PNA resulted in 100-fold decrease in assay detection limit as compared with the PNA method alone. Clearly, this improvement will contribute to more accurate molecular diagnosis of MAS.

In the present study, 56% of MAS patients were positive for a *GNAS* mutation by the PNA method. Similar mutation detection rate with the nested PCR method or the PNA method have been reported [9]–[11]. Of clinical importance, we could detect *GNAS* mutations by the PNA-NGS method in three out of seven ‘PNA-negative’ MAS patients. This would be not only due to ultra-deep sequencing of NGS, but also due to nature of the mutation detection methods (qualitative *vs* quantitative), because a patient with relatively high RMA (Patient 4 in **Table 1**) was missed by PNA. Considering that mutation detection rate is more than 90% when affected tissue was available [10], we believe that improvement of mutation detection by the PNA-NGS approach is due to increase of true positives, although we cannot discriminate true positives from false positives in the present study. Future studies using paired PBL-affected tissue(s) samples, which have discordant test results (*e.g.,* negative in PBL but positive in affected tissue(s)) will clarify the true diagnostic performance of the PNA-NGS approach.

In summary, we successfully developed an NGS-based mutation detection method for MAS, allowing quantitative and sensitive molecular diagnosis. The PNA-NGS method achieved 100-fold decrease in assay detection limit, as compared with the PNA method. Our study exemplifies the utility of NGS-based approaches to diagnose congenital disorders due to low-abundance somatic mutations from peripheral blood.
